# Data on Swiss grapevine growers’ production, pest management and risk management decisions

**DOI:** 10.1016/j.dib.2023.109652

**Published:** 2023-10-08

**Authors:** Lucca Zachmann, Chloe McCallum, Robert Finger

**Affiliations:** Agricultural Economics and Policy Group, ETH Zurich, Switzerland

**Keywords:** Viticulture, Fungus-resistant, Grapevines, Pest management, Pesticide use, Sustainable agriculture, Risk management

## Abstract

We present survey data from 436 grapevine growers across Switzerland and their production, pest, and risk management decisions. The online survey was conducted in spring 2022 in the three main official languages in Switzerland (German, French, Italian). The survey was used to obtain information on variety choice and farm management strategies, as well as farmer, farm, and spatial environmental characteristics. Moreover, we collected information around fungus-resistant grapevine varieties such as knowledge, attitudes, and perceptions of these varieties. We also elicited the current cultivation and growers’ intentions on future acreage under these varieties. In addition, data were collected on growers’ pest management strategies against weeds, insects, and fungi. Characteristics of the farm manager collected include education, farming goals, wine-related expertise, and information sources used. Information about the farm consist of marketing channels, labels, direct payment schemes, production systems and pesticide application machinery, among other details. Moreover, risk and time preferences, self-efficacy and locus of control were collected via self-assessed scales. The survey data were matched with spatial climatic data on municipality level (e.g. on temperature, precipitation, the number of yearly hail days, average sunshine duration and relative humidity) as well as pest pressure (e.g. infection risk by *Oidium* and *Peronospora viticola*) at weather station level.

Specifications Table

**Table:** 

Subject	Agricultural Economics
Specific subject area	Adoption of fungus-resistant grapevines, pest management, risk preferences, risk perceptions
Type of data	CSV file (semicolon delimited), XLSX
How the data were acquired	Online survey using LimeSurvey combined with meteorological data from weather stations and gridded climate datasets.
Data format	Raw Partly filtered (for reasons of confidentiality) Partly cleaned
Description of data collection	The online questionnaire was distributed in German, French and Italian via LimeSurvey to a sample of 2’346 grapevine growers in Switzerland from January 13th to April 8th 2022. A total of 436 farmers responded completely to the survey (response rate: 18.6%). Participation was incentivized. The data were anonymized.
Data source location	• Institution: ETH Zurich • City/Town/Region: Zurich • Country: Switzerland
Data accessibility	Repository name: ETH Zürich Research Collection Direct URL to data: https://doi.org/10.3929/ethz-b-000568595.
Related research article	Zachmann, Lucca, Chloe McCallum, and Robert Finger. “Nudging Farmers towards Low‐pesticide Practices: Evidence from a Randomized Experiment in Viticulture.” Journal of the Agricultural and Applied Economics Association 2, no. 3 (July 31, 2023): 497–514. https://doi.org/10.1002/jaa2.76.

## 1 Value of the Data


 

• Farm-level data collection specifically about the adoption of and perceptions about fungus-resistant grapevine varieties, which can substantially contribute to pesticide risk reduction in viticulture without compromising yields.
• Increasing pest pressures in vineyards make this data vital for other countries to understand the adoption of fungus-resistant grapevine varieties.
• Researchers, policy makers, and food-value chain actors can use the data to understand barriers and determinants of the adoption of fungus-resistant grapevine varieties.
• On-farm pest management decisions are influenced by environmental conditions; here presented survey data is thus matched with secondary data on temperature, precipitation, number of yearly hail days, average sunshine duration and relative humidity as well as with fungal pest pressure information (i.e. infection risk by *Oidium* and *Peronospora viticola*)
• The data on production choices, pest management and risk management strategies used in grapevine production as well as the treatment experiment, combined with extensive collection of data about farmer and farm characteristics as well as behavioral characteristics, allows for standalone research and comparison with other studies as well as merging with complementing databases. Moreover, the data can be used in meta-analyses and replication studies.

## 2 Data Description

**Fig. 1:**
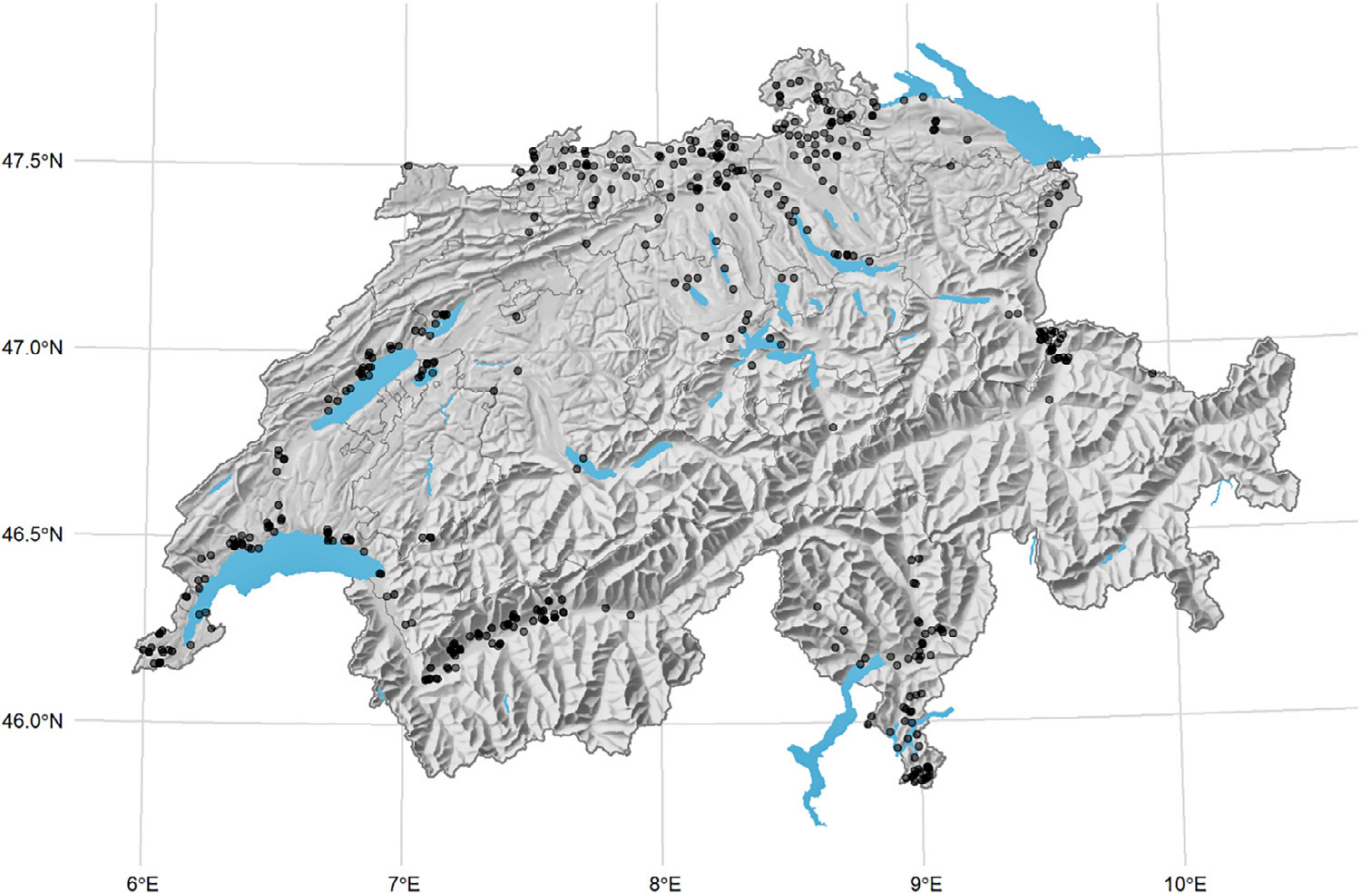
Sample overview. *Note:* Scatters in darker colors refer to more than one observation in a municipality. The scatters are randomly positioned within municipalities and do not represent actual locations of the farms. This is done for confidentiality reasons.

We collected survey data from 436 grapevine growers in Switzerland about their production, pest management and risk management decisions. We asked growers about their current pest management practices and future plantation expectations. We particularly focused on pest management, the uptake of fungus-resistant grapevine varieties, and farm and farmer characteristics. The data collection was carried out with an online survey from January 13th to April 8th 2022 in German, French and Italian.^1^ Refer to Fig. 1 for an overview of the sample. The dataset, survey and codebook describing the variables are available online on the ETH Zürich Research Collection: https://doi.org/10.3929/ethz-b-000568595.

In the survey, we collected information related to fungus-resistant grapevine varieties including growers’ current and intended future cultivation, their perceptions and knowledge related to fungus-resistant varieties and information sources they use. This is relevant because fungus-resistant grapevines are the most effective strategy to reduce pesticide use in viticulture without compromising yields [2]. Furthermore, a wide range of pest management strategies (e.g. pest control strategies against weeds, insects, and fungi, and pesticide use) were collected. Farm (e.g. size, production system, focus) and farmer (e.g. age, gender, education) characteristics were also collected. Moreover, risk preferences in four domains (production, agriculture, marketing and plant protection) and time preferences were elicited using self-assessment questions [following 3,4]. Finally, data collected also include information on locus of control and self-efficacy. The data collection builds on previous surveys described in Knapp, Bravin, and Finger [5].

The collected data covers 2’112.2 hectares of acreage under grapevines, representing 14.4% from the total cultivation area in Switzerland (see Table 1).

**Table 1:** Sample representativeness in terms of observable characteristics.

	Sample of grapevine growers	Switzerland (whole farming population)	Source
**Farmer characteristics**			
Age	66% of farms in our sample were managed by people over 50 years of age	55% of farms in Switzerland were managed by people over 50 years of age	[6]
Female farmers	9%	6%	[7]
**Farm characteristics**			
Organic producers	15%	15%	[7]
Farm size (in ha)	8.7	21.2	[7]
**Grapevines**			
Land under grapevines (in ha)	2’112.2 (14%)	15’038	[6]
Share of fungus-resistant varieties	4.9%	3%	[6]
Share of red varieties	57%	56%	[6]

*Note:* The table provides characteristics of observable variables of our sample comparing mean values for Swiss agriculture at large, depicting representative values for the relevant statistics. For instance, the share of organic producers, the share of fungus-resistant varieties and variety color our sample matches closely with the Swiss population of farmers at large. The sample slightly overrepresents older and female growers, and includes smaller farms compared with national statistics. Note, however, that vineyards or specialty crop farms in general are typically smaller compared to farms specialized in arable crop production.

**Fig. 2:**
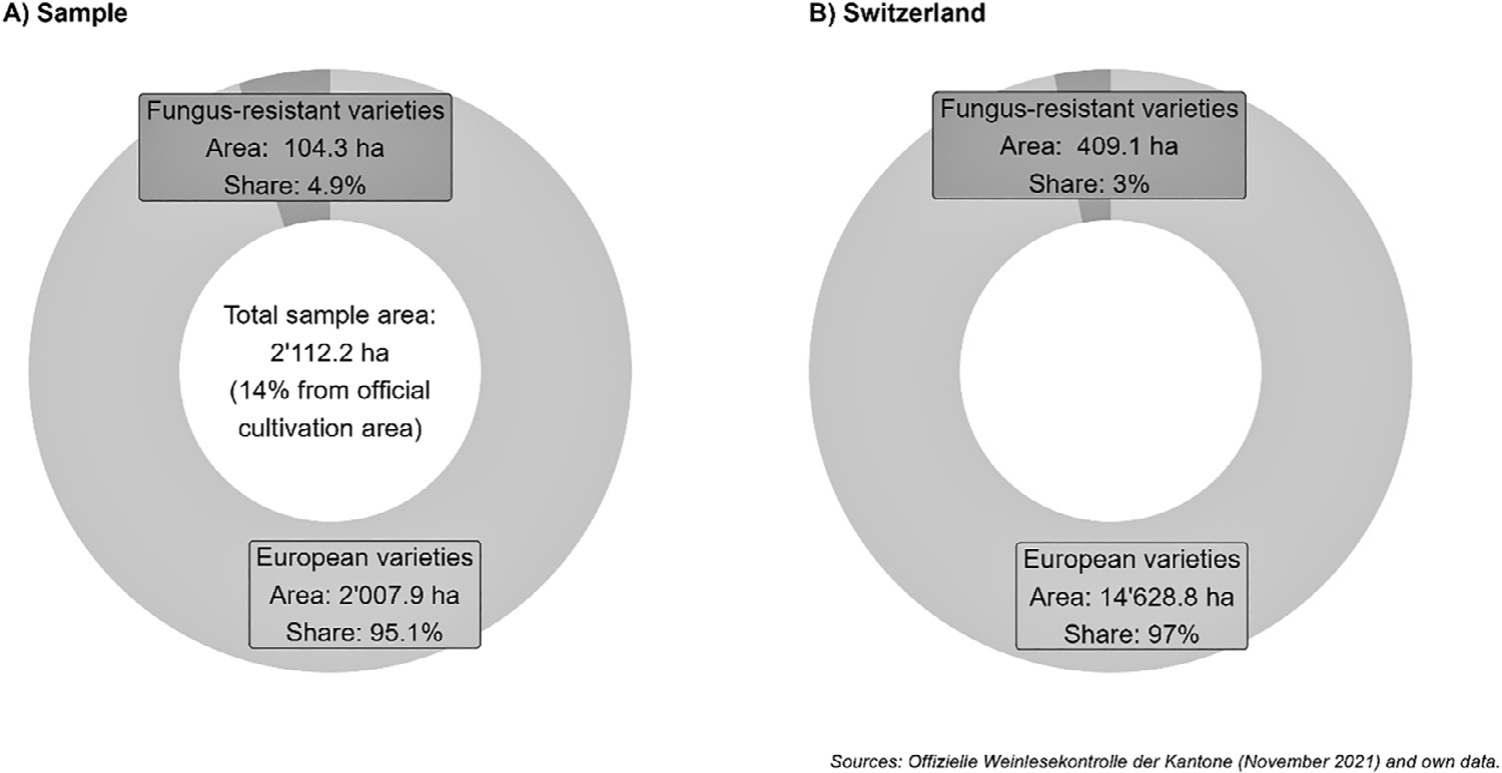
Sample representativeness in terms of variety types (Fungus-resistant vs. European varieties).

Fig. 2 shows sample representativeness in terms of variety types (i.e. areas devoted to fungus-resistant and European varieties). The figure shows that our survey is representative in terms of land devoted to fungus-resistant varieties vis à vis European varieties, with a share of land devoted to fungus-resistant varieties of 4.9%, while official cultivation data is 3% [8].

We combined survey data with meteorological data from 102 weather stations across Switzerland. The matching of farms participating in the survey and weather stations was based on a farm's zip code, i.e. taking the closest weather station for each farm. More specifically, we used daily data from Agrometeo [10] from the years 2012 to 2021 which is one year prior to survey data collection.^2^ We constructed yearly average values for temperature, precipitation and relative humidity. To infer to fungal disease pressure, we also match each record with *Oidium* (powdery mildew) and *Peronospora viticola* (downy mildew) infection risk indices [9]. Moreover, we calculated yearly summations for the daily *Oidium* and *Peronospora viticola* risk indices to measure overall yearly pest pressure. The indices calculate infection risk from *Oidium* and *Peronospora viticola*, respectively, based on meteorological conditions (temperature, precipitation, and relative humidity) and the ontogenic resistance of the bunches to infection [9]. For example, the organs and tissues of the grapevines have different infection sensitivity during their development stages. Therefore, infection risk is highest in June/July and decreases towards harvest (end of August to October, depending on year, variety, and location). Note that the indices have different units. While *Oidium* infection risk is measured in percentages, i.e. from 0 (no infection risk) to 100% (high infection risk), the *Peronospora viticola* risk index uses a categorical scale, i.e. 1 (no infection risk), 2 (medium infection risk), and 3 (high infection risk). We matched the station data to our sample by minimizing the distances between the observations and weather stations (i.e. the 'great-circle-distance') according to the haversine method. Moreover, we aggregated gridded datasets on the mean yearly hail days from 2002 until 2022 and the average sunshine duration from March to September from 1991 to 2020 relative to the maximum possible (in %) to the zip code level [11,12] (see Appendix A for more information).^3^

## 3 Experimental Design, Materials and Methods

We used LimeSurvey, an online platform, to design and carry out our survey. The survey was pre-tested with 4 grapevine experts and a pilot study was completed with 13 grapevine growers. After the feedback was incorporated, the survey was translated into three languages: German, French and Italian. Translations were validated by native speakers. As an incentive to participate in the main survey, we indicated that 25 vouchers (for a dealer of agricultural goods in Switzerland) with a value of 50 Swiss Francs (CHF) each could be won by participants who completely answered to the questionnaire. Additionally, participants could opt in to receive individual feedback on the survey results if they were interested. This feedback included aggregate information on current grapevine production practices and individual feedback about future expectations of the participants relative to their peers.

The median time to complete the survey was 32 minutes. There were 65 questions in the survey divided into the following sections:

(i) Information on all grapevine varieties grown, and their cultivated areas
(ii) Agronomic and pest management practices
(iii) Farmer characteristics
(iv) Farm characteristics
(v) Perceptions and knowledge about fungus-resistant varieties
(vi) Behavioral characteristics
(vii) Information treatment experiment

## 4 Grapevine Varieties and Cultivated Areas

At the start of the questionnaire, we asked survey participants about their farm size (in Are, i.e. 100s of square meters). Thereafter, we elicited from a multiple-choice menu of the 35 most frequently planted grapevine varieties in Switzerland which varieties the survey participants grow on their farm.^4^ Moreover, in case the participant has adopted another variety that was not listed in the menu, we provided the option to add up to 15 more varieties manually. For each variety, we asked for the area under cultivation to receive a complete picture on the variety portfolio of a farm. We follow the Federal Office for Agriculture [8] to identify fungus-resistant grapevine varieties. Overall, we identified 143 different grapevine varieties used by the participants, of which 51 are fungus-resistant varieties (see Appendix B).

## 5 Agronomic and Pest Management Practices

We also asked growers about their on-farm pest management practices. Specifically, we surveyed an extensive list of employed pest management practices against weeds, insects, and fungi. The practices included preventive, biological, technology-based, and chemical strategies. These practices were elicited also accounting for growers’ voluntary participation in direct payments (e.g. whether they participate in programs on not using herbicides).^5^ Moreover, we elicited whether growers applied particularly environmentally toxic fungicides by including a list of ten product classes containing quasi-perfect substitutes of 22 fungicides overall. The included products made up 5% from all listed fungicides allowed to be used in Swiss vineyards. The pesticide load indicator was used to assess the toxicity of the fungicides (see [13]). Products allowed to be used in organic and non-organic viticulture were included. Refer to section “Information treatment experiment” for more information.

## 6 Farmer Characteristics

Farmer-specific information obtained included participants’ gender, year of birth, educational background, self-assessed expertise in grapevine production, vinification, and marketing. Additionally, we also elicited what tasks the survey participant completes at the vineyard (e.g. field work, plant protection, office work, planting decisions, investment decisions, and vinification).

We used a binary question to elicit whether growers had looked for information about fungus-resistant grapevines. In the affirmative case we provided ten multiple-choice options where information was sought and in the negative case four multiple-choice options why no information was sought. We also asked where (i.e. at which information sources) growers search for plant protection information more generally.

## 7 Farm Characteristics

Since the adoption of fungus-resistant grapevine varieties is currently small, and the adoption process has multiple stages, we elicited what stage of adoption the farms are in. We included answer options following the adoption stages described in Weersink and Fulton [14]. The different stages are the awareness of these varieties, the evaluation of their overall potential, the adoption of at least one variety, and the potential dis-adoption. Additionally, if a participant has indicated that they have evaluated the potential, we queried the assigned potential for red and white fungus-resistant varieties on a Likert scale from 0 (no potential) to 5 (great potential).

Additional farm characteristics included machinery used to apply plant protection products, such as hand sprayers, low drift nozzles, tunnel recycling sprayers but also drones and helicopters. We also asked participants whether the farm has taken part in one of the four different direct payment schemes for the partial or complete abandonment of herbicides and fungicides. Moreover, we elicited whether they received direct payments for purchases/investments of low-drift application machinery.

Our questionnaire also included a rich set of marketing characteristics, i.e. how grapevines and/or wine produced on the farm is marketed. We asked survey participants about their marketing arrangements for grapevines and/or wine. More specifically, we elicited the percentages of produce they market as grapevines to winemakers, cooperatives, or commerce, or as wine to commerce, major distributors, gastronomy or directly to consumers. Furthermore, we include relevant labels or terms used to market wine as well as whether geographical denominations (e.g. Appellation d'Origine Contrôlée or Designation of Origin) are used at the variety level.^6^

Standard variables describing farm characteristics are the employed labor on the farm, share of farmland that is leased, farm strategy, diversification, and specialization, information sources, and the share of income from agriculture and viticulture [5].

## 8 Perceptions and Knowledge about Fungus-Resistant Varieties

Our survey contained a wide range of perceptions of and knowledge about fungus-resistant grapevine varieties. For instance, we asked growers about their self-reported knowledge about fungus-resistant grapevines on a 6-points Likert scale from knowing nothing (0) to very knowledgeable (5). Moreover, we asked the grapevine growers by what percentage they think fungus-resistant grapevines reduce the use of fungicides in Switzerland. Answer options ranged from nothing (0%) to no fungicide needed anymore (100%) with intervals in between.

We also elicited growers’ perceptions about fungus-resistant varieties in comparison to traditional varieties. The considered perceptions were with respect to quality, marketing difficulty, willingness-to-pay from consumers, future use, environment benefits, and human health benefits for growers and communities surrounding farms. For each of these comparisons, participants were asked to choose an answer option on a 5-point scale ranging from strongly disagree to strongly agree.

We elicited survey participants’ future perceptions about viticulture in general and fungus-resistant varieties in particular. Our future reference was ten years for two reasons. First, a vineyard's average life is approximately 25-35 years [15]. Second, human cognitive ability is bounded, making it increasingly difficult to accurately project far into the future [16]. We therefore used ten years as a trade-off. Along these lines, we asked growers what share of their viticultural land will be replanted in ten years (in percentage). We also asked how much of their land will be devoted to fungus-resistant varieties in ten years (see Information treatment experiment). Moreover, we also surveyed general questions regarding the state of viticulture in Switzerland in ten years. For instance, we asked whether growers expect to be still working in viticulture in ten years, whether they expect to grow grapevines according to organic standards, or whether they believe new technologies will allow reduced fungicide use. Additionally, we elicited whether growers expect that weather events will increase cryptogamic pressure, whether copper will be a banned substance, or whether fungicide resistance will be a large issue.

We also asked growers about their perceptions on the effect of plant protection products on wine quality, wine quantity, soil, the environment, and on growers’ health. Additionally, we included questions on the perceived importance of (farm) biodiversity, and whether biodiversity decline is considered an issue for production. Moreover, we asked whether growers use greening of inter-row space at their farm and if they consider or use (flowering) species-rich seed mixtures for greening inter-row space.

## 9 Behavioural Characteristic

We also added questions on non-cognitive skills, i.e. locus of control [17] and self-efficacy [18] to our questionnaire as these concepts are relevant to explain growers’ pest management decisions [19]. We phrased our questions regarding self-efficacy similar to Knapp et al. [5] and those for locus of control based on Abay et al. [20]. Therefore, locus of control and self-efficacy were measured with 5-points Likert scales and related to grapevine/wine production. We included seven questions overall, three on locus of control and four questions on self-efficacy with answer options ranging from strongly disagree to strongly agree.

Moreover, we elicited time and risk preferences that are expected to explain decisions by growers [e.g. 21]. For time preferences, we followed Falk et al. [4] and asked growers how willing they are to give up income that is beneficial for them or their farm today in order to benefit more from that in the future. We used a 11-points Likert scale, ranging from not willing to very willing to give up income. For risk preferences, growers were asked on an 11-points Likert scale how willing they were to take or mitigate risks in the areas of production, market and prices, plant protection, and agriculture in general, respectively [3,5].

## 10 Information Treatment Experiment

Our survey also included a pre-registered information treatment experiment (see Zachmann et al. [1]).^7^ In the experiment, we first elicited growers’ expectations about their share of land devoted to fungus-resistant grapevine varieties in ten years (prior elicitation). We followed Hardaker [22] to elicit a triangular distribution of this share to account for uncertainty with respect to the future projection. Consequently, we asked participants about the most likely share of land they will devote to fungus-resistant grapevines in ten years, as well as the smallest and largest possible shares.

Next, we asked participants if they used selected fungicides in the last growing season. The fungicide products we surveyed included the most toxic products allowed in Swiss viticulture regarding their load on the environment. In Switzerland, 403 fungicides with unique names are registered for use in vineyards. For all registered fungicides, we calculated the environmental fate and ecotoxicity load [13,23]. Subsequently, we identified products which are highly toxic, defined as having a joint environmental fate and ecotoxicity load that is in the top 95th percentile of the environmental load distribution. In the survey software (i.e. LimeSurvey), we asked participants how many products out of ten clusters with the most toxic products they used in the last growing season. Subsequently, we randomly provided participants either with personalized, general or no information as displayed in Table 2.

**Table 2:** Information treatments.

Information group	Displayed feedback
Personalized	Based on a scientific risk assessment for plant protection products which considers environmental side-effects (i.e. persistence in soil / biomass and toxicity to non-target organisms from the Pesticide Property Database) of the active substances in a product and its formulation, 22 fungicides (5% from all fungicides registered for use in grapevines in Switzerland) are considered highly toxic to the environment. *Based on the your previous answers, you use [#of_products] out of these fungicides*.
General	Based on a scientific risk assessment for plant protection products which considers environmental side-effects (i.e. persistence in soil / biomass and toxicity to non-target organisms from the Pesticide Property Database) of the active substances in a product and its formulation, 22 fungicides (5% from all fungicides registered for use in vines in Switzerland) are considered highly toxic to the environment.
Control	-

After the information intervention, we elicited the expected share again but carefully reformulated the main question in the posterior elicitation to mask the ultimate purpose of the experiment and avoid experimenter demand effects while maintaining comparability of the two questions.

## Ethics statements

The survey has been approved by the Ethics Commission of ETH Zurich on October 27 2021 with confirmation number 2021-N-175. All participants declared informed consent prior to entering the survey.

## CRediT Author Statement

**Lucca Zachmann**: Conceptualization, Methodology, Writing - original draft preparation, Visualization, Data Preparation, Data Analysis. **Chloe McCallum**: Conceptualization, Methodology, Writing - Reviewing/Editing. **Robert Finger**: Conceptualization, Methodology, Writing - Reviewing/Editing, Funding.

## Data Availability

Data on Swiss grapevine growers’ production, pest management and risk management decisions (Original data) (ETH Zürich Research Collection)

## Appendix Supplementary materials

Image, application 1

## Footnotes

1 We contacted the farmers either in German, French or Italian (the main official languages in Switzerland) based on the main language spoken in their municipality. We shared email invitations with 2’346 grapevine growers who had previously taken part in surveys and/or were registered on SwissWine, the online platform of the Swiss Vine and Wine Association (see details below and in Zachmann et al. [1]). We thus followed a convenience non-random sampling approach. Refer to Appendix C for the survey questionnaire.

2 Agrometeo provides weather data from over 180 stations to predict crop disease and pest risks, offering decision support for optimized agricultural protection measures: https://www.agrometeo.ch/de.

3 March to September is roughly the time period during which vines have leaves and thus sunshine is an important factor for their development.

4 We asked participants to only mention the varieties with a cultivated on an area over or equal to 1 are.

5 See e.g. the website of the Swiss Federal Office of Agriculture https://www.blw.admin.ch/blw/de/home/instrumente/direktzahlungen.html.

6 The included labels are: organic, demeter, IP Suisse, Delinat, Vinatura. The included terms are PIWI and natural wine.

7 Refer to https://aspredicted.org/TL3_C7G for the pre-registration document.
