# Supplementary material for: Data on Swiss grapevine growers’ production, pest management and risk management decisions
Source: Data Brief. 2023 Oct 8;51:109652. doi: 10.1016/j.dib.2023.109652 (PMC10577060; doi:10.1016/j.dib.2023.109652)
Supplement: Supplementary file 1 [file mmc1.docx]

**Appendix**

**A Environmental variables**

This section contains information on the environmental data that we match to our survey. First, we match the yearly average number of hail days during the summer half-year (i.e. from April to September) at the municipality level to the sample. The size is derived from radar measurements. A hail day is defined as a day on which a high probability of hail on the ground was concluded from the radar measurements. The 24 hours between 06 UTC and 06 UTC of the following day are considered. More information here: <https://doi.org/10.18751/Climate/Griddata/CHHC/1.0>

**Figure A1**: Mean yearly hail days at municipality level

**
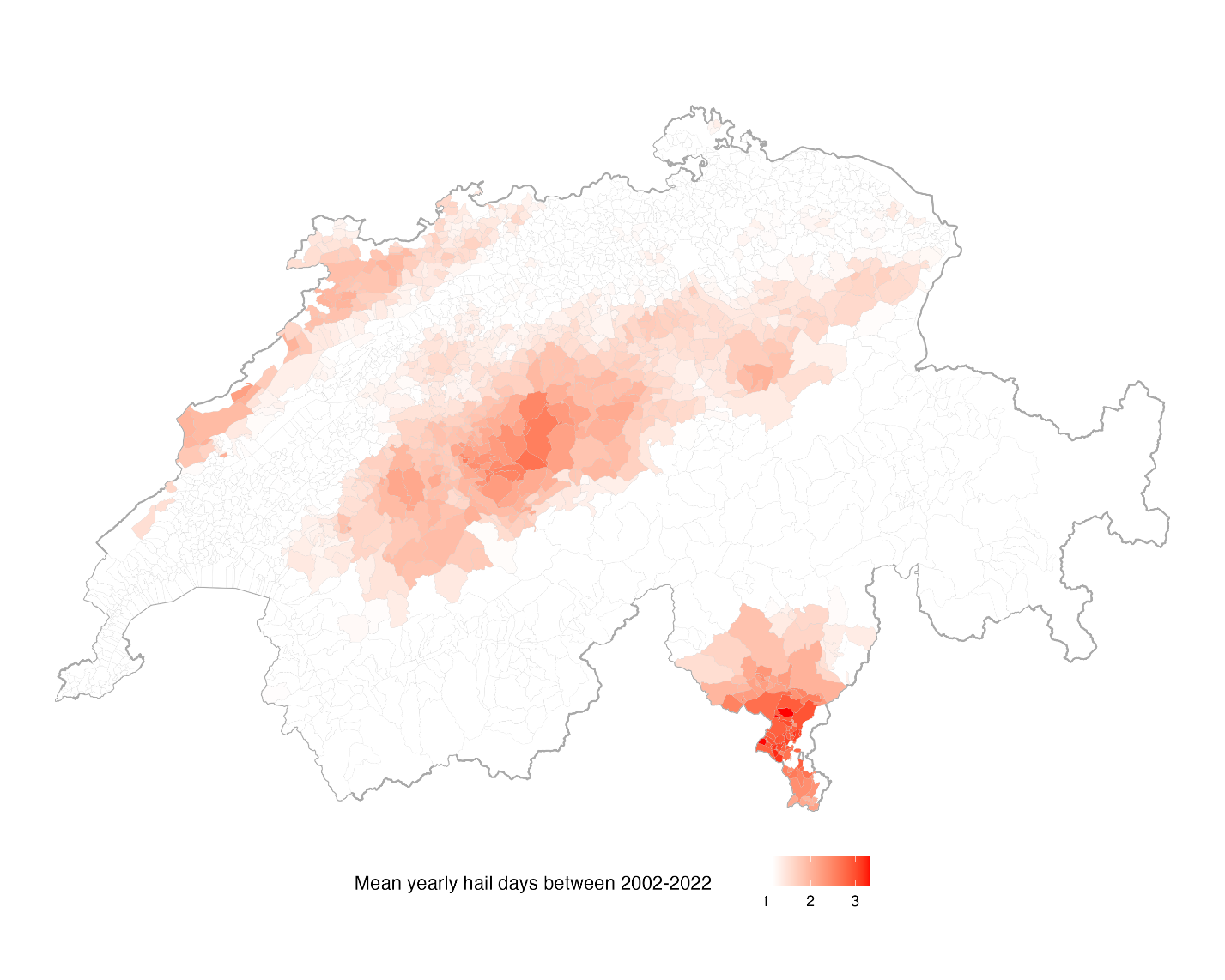
**

Second, we match the mean average sunshine duration from March to September from 1991 to 2020 relative to the maximum possible (in %) at the municipality level to the sample. More information can be here: <https://opendata.swiss/de/dataset/klimanormwerte-sonnenscheindauer-1961-1990>

**Figure A2**: Mean sunshine duration from March do September at municipality level

**
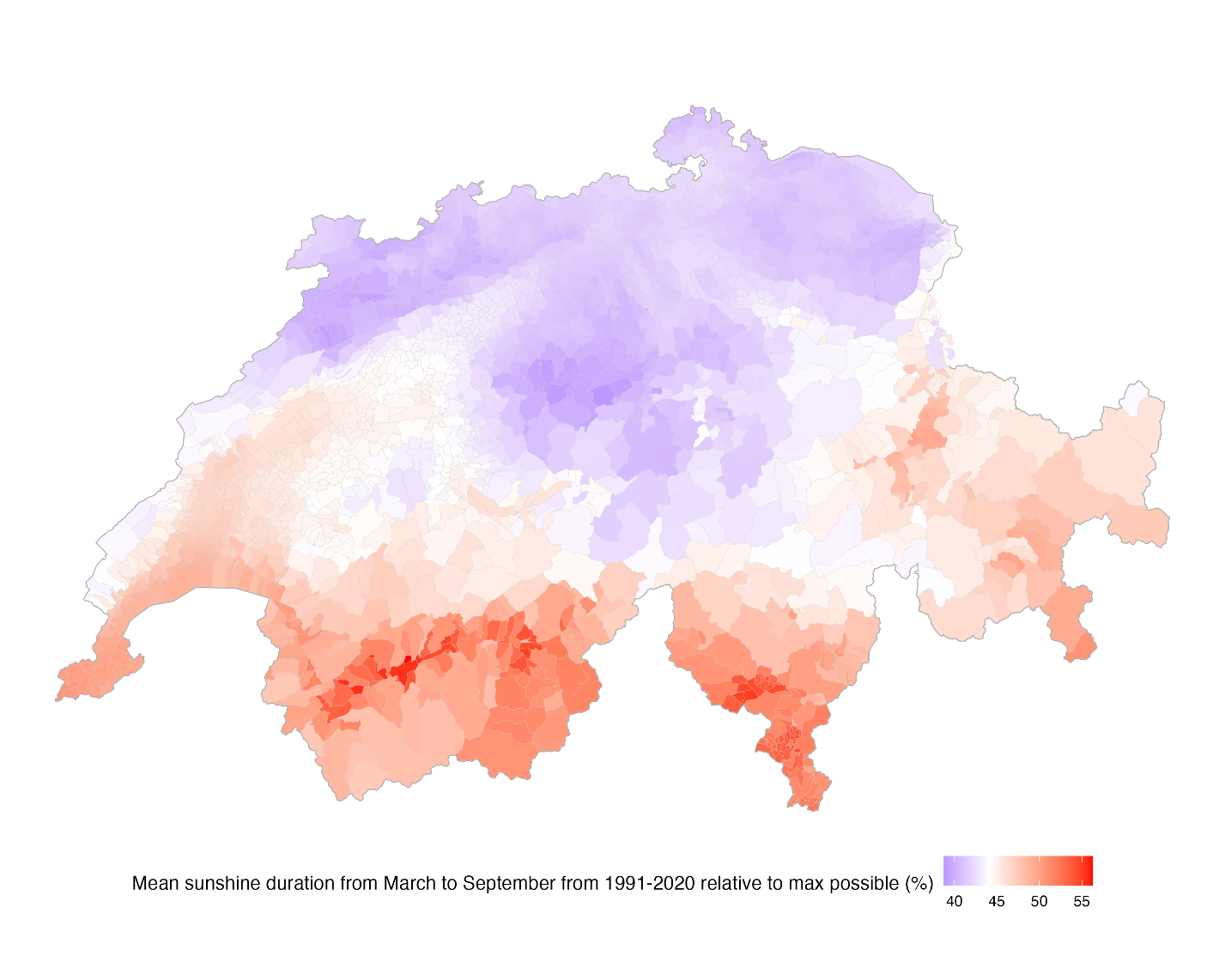
**

Last, we merge pest infection risk data from 102 weather stations across Switzerland to the sample (data comes from [www.agrometeo.ch](http://www.agrometeo.ch)). We use *Oidium* (powdery mildew) and *Peronospora viticola* (downy mildew) infection risk indices. We calculate average values and yearly sums for the *Oidium* and *Peronospora viticola* risk indices to measure aggregate and overall pest pressure per year (averaged between 2012 and 2021), which are based on meteorological conditions and bunch ontogenic resistance. The indices evaluate infection risk from *Oidium* and *Peronospora viticola*, respectively, and have different units. *Oidium* infection risk is expressed as a percentage, ranging from 0 to 100%, while the *Peronospora viticola* risk index uses a categorical scale of 1 (no infection risk), 2 (medium infection risk), and 3 (high infection risk). We matched station data to our sample using the haversine method to minimize the distances between municipality centroids from the observations and weather stations.

**Figure A3**: Average infection risk from downy mildew at weather station level


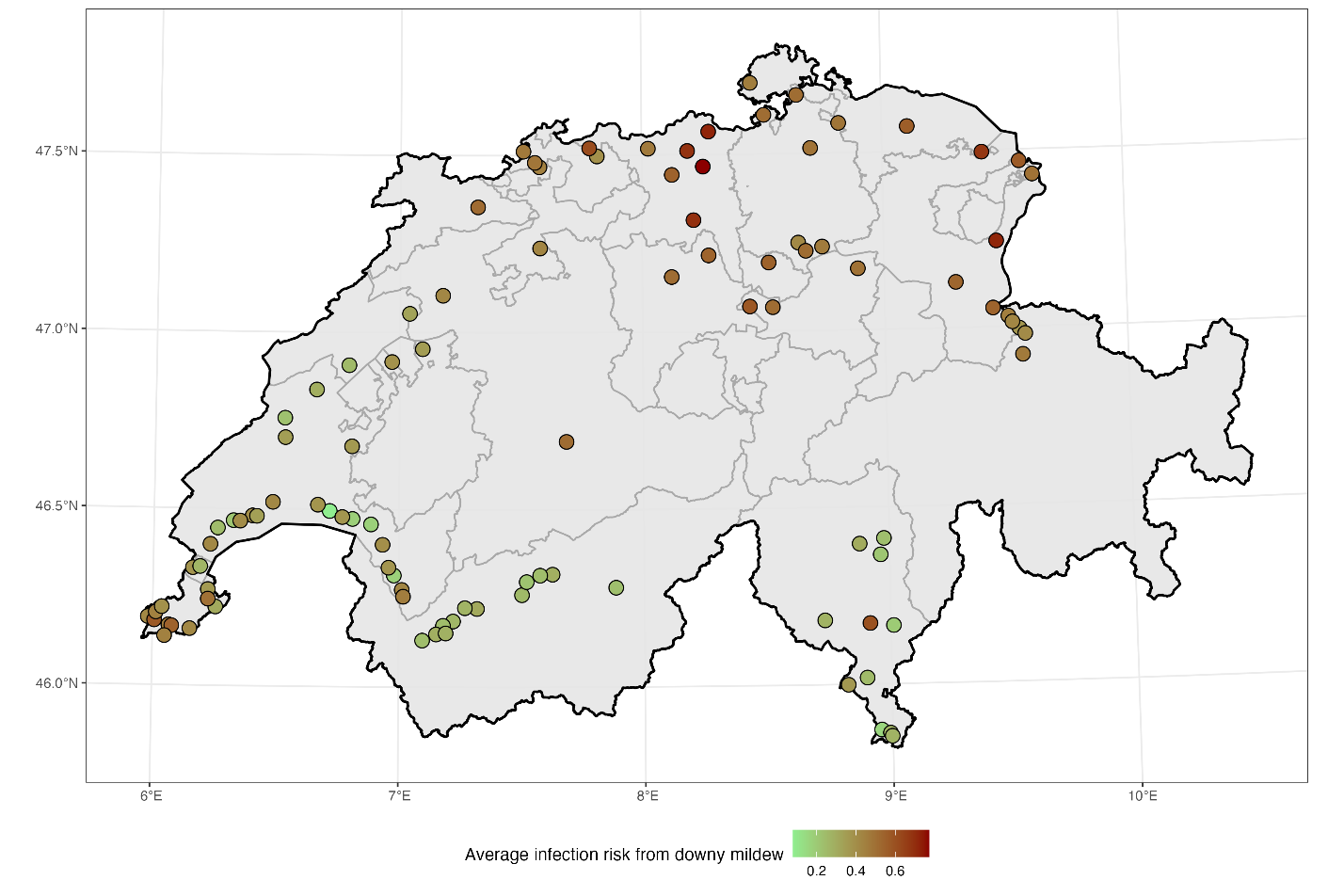


**B Grapevine varieties**

Below we provide information on identified grapevine varieties, their type, color and devoted cultivation area. The classification into fungus-resistant and European varieties as well as variety color follows [8].

**Table B1**: Variety identification according to type, color, and devoted area

| **Grapevine variety** | **Variety type** | **Variety color** |
| --- | --- | --- |
| Acolon | European | Red |
| Aligote | European | White |
| Altesse | European | White |
| American hybrids | European | Red |
| Amigne | European | Red |
| Ancelotta | European | Red |
| Arinarnoa | European | Red |
| Auxerrois | European | White |
| Bacchus | European | White |
| Baco noir | Fungus-resistant | Red |
| Barbera | European | Red |
| Birstaler muscat | Fungus-resistant | White |
| Blaufraenkisch lemberger | European | Red |
| Bluetenmuskateller | Fungus-resistant | White |
| Bondola | European | Red |
| Bronner | Fungus-resistant | White |
| Cabernello | Fungus-resistant | Red |
| Cabernet blanc | Fungus-resistant | White |
| Cabernet cantor | Fungus-resistant | Red |
| Cabernet carol | Fungus-resistant | Red |
| Cabernet cortis | Fungus-resistant | Red |
| Cabernet cubin | European | Red |
| Cabernet dorsa | European | Red |
| Cabernet eidos | Fungus-resistant | Red |
| Cabernet franc | European | Red |
| Cabernet jura | Fungus-resistant | Red |
| Cabernet noir | Fungus-resistant | Red |
| Cabernet sauvignon | European | Red |
| Cabertin | Fungus-resistant | Red |
| Carminoir | European | Red |
| Chardonnay | European | White |
| Charmont | European | White |
| Chasselas | European | White |
| Chenin blanc | European | White |
| Completer | European | White |
| Cornalin | European | Red |
| Cornarello | European | Red |
| Dakapo | European | Red |
| Diolinoir | European | Red |
| Divico | Fungus-resistant | Red |
| Divona | Fungus-resistant | White |
| Donauriesling | Fungus-resistant | White |
| Doral | European | White |
| Dornfelder | European | Red |
| Dunkelfelder | European | Red |
| Durize | European | Red |
| Elbling | European | White |
| Ferradou | European | Red |
| Freisamer freiburger | European | White |
| GF 48 12 | Fungus-resistant | White |
| Galotta | European | Red |
| Gamarello | European | Red |
| Gamaret | European | Red |
| Gamay | European | Red |
| Garanoir | European | Red |
| Gewuerztraminer | European | White |
| Glera | European | White |
| Grenache | European | Red |
| Gruener veltliner | European | White |
| Helios | Fungus-resistant | White |
| Humagne blanc | European | White |
| Humagne rouge | European | Red |
| IRAC 1933 | Fungus-resistant | Red |
| Johanniter | Fungus-resistant | White |
| Kerner | European | White |
| Lagrein | European | Red |
| Laurot | Fungus-resistant | Red |
| Leon millot | Fungus-resistant | Red |
| Malbec | European | Red |
| Mara | European | Red |
| Marechal foch | Fungus-resistant | Red |
| Marechal foch leon millot | Fungus-resistant | Red |
| Marsanne blanche | European | White |
| Marselan | European | Red |
| Merello | European | Red |
| Merlot | European | Red |
| Monarch | Fungus-resistant | Red |
| Mondeuse rouge | European | Red |
| Mourvedre | European | Red |
| Mueller thurgau | European | White |
| Muscaris | Fungus-resistant | White |
| Muscat | European | White |
| Muscat bleu | Fungus-resistant | White |
| Muscat oliver | European | White |
| Muscatin | European | White |
| Nebbiolo | European | Red |
| Nerolo | European | Red |
| Nobling | European | White |
| Orion | Fungus-resistant | White |
| Persan | European | Red |
| Petit manseng | European | White |
| Petit meslier | European | White |
| Petit verdot | European | Red |
| Petite arvine | European | White |
| Pinorico | European | Red |
| Pinot blanc | European | White |
| Pinot gris | European | White |
| Pinot meunier | European | Red |
| Pinot noir | European | Red |
| Pinot nova | Fungus-resistant | Red |
| Pinotage | European | Red |
| Pinotin | Fungus-resistant | Red |
| Plant robert | European | Red |
| Prior | Fungus-resistant | Red |
| Rauuschling | European | White |
| Regent | Fungus-resistant | Red |
| Reze | European | White |
| Rheinriesling | European | White |
| Rondo | Fungus-resistant | Red |
| Roter mueller thurgau | European | Red |
| Saint laurent | European | Red |
| Saphira | Fungus-resistant | White |
| Satin noir | Fungus-resistant | Red |
| Sauvignon blanc | European | White |
| Sauvignon soyhieres | Fungus-resistant | White |
| Sauvitage | Fungus-resistant | White |
| Savagnin blanc | European | White |
| Scheurebe | European | White |
| Semillon | European | White |
| Servagnin | European | White |
| Seyval blanc | Fungus-resistant | White |
| Siegerrebe | European | White |
| Solaris | Fungus-resistant | White |
| Souvignier gris | Fungus-resistant | White |
| Sylvaner | European | White |
| Syrah | European | Red |
| Tannat | European | Red |
| Tempranillo | European | Red |
| Triomphe d alsace | Fungus-resistant | Red |
| Trousseau | European | Red |
| VB cal 1 14 | Fungus-resistant | Red |
| VB cal 1 15 | Fungus-resistant | Red |
| VB cal 1 20 | Fungus-resistant | Red |
| VB cal 1 21 | Fungus-resistant | Red |
| VB cal 1 22 | Fungus-resistant | Red |
| VB cal 1 28 | Fungus-resistant | Red |
| VB cal 1 36 | Fungus-resistant | Red |
| VB cal 6 04 | Fungus-resistant | Red |
| VB cal 82 6 | Fungus-resistant | Red |
| Vidal blanc | Fungus-resistant | White |
| Viognier | European | White |
| Zinfandel primitivo | European | Red |
| Zweigelt | European | Red |

**C Survey questionnaire**

*Ce sondage est également disponible en français (veuillez choisir ci-dessus). Questo sondaggio è disponibile anche in italiano (selezionare sopra). Diese Umfrage ist auch in Deutsch verfügbar (bitte oben auswählen).*

**Dear grapevine growers**

We are pleased that you are taking part in this survey on your current and future viticultural practices and perceptions. The survey will provide important insights for agricultural practices, extension services and research.

The aim of the study is to find out which factors influence the decision of the farmers' grapevine planting decisions including fungus-resistant varieties, management choices, pest management strategies, and distribution channels.

The survey will only take about 20-30 minutes.

We will also raffle 25 prizes of Landi vouchers worth 50 CHF among all participants who have fully answered the survey.

**Thank you very much for your participation!**

Best regards,

XXXXX

If you have any questions, please contact:

XXXXX
XXXXX

XXXXX

**Administration:**

A1*. Your participation in the survey is voluntary. Your data and information will of course be treated with strict **confidentiality** and used **anonymously** for **scientific** purposes. I agree to the information and the conditions of participation and data protection that can be found here.

I hereby confirm that my participation is voluntary and that my data may be used

A2*. Would you like to receive the results of the survey? We will send you an individual evaluation.

Yes

No

A3*. Would you like to participate in the lottery of 25 Landi vouchers valued at CHF 50 each?

Yes

No

**Section 1: Grape varieties**

C1*. What is your total farmland in **are**? __________

C2*. Which grape varieties do you have on your vineyard? Please only mention the varieties with a cultivated area over or equal to **1 are**.

*Hint: Tick if you grow this variety. Varieties that are not listed can be added manually at the end.*

*Please specify all grown varieties on your vineyard.*

Amigne

Cabernet Dorsa

Cabernet Franc

Cabernet Jura

Cabernet Sauvignon

Chardonnay

Chasselas / Gutedel

Cornalin / Landroter

Diolinoir

Divico

Doral

Galotta

Gamaret

Gamay

Garanoir

Gewürztraminer

Humagne Blanc

Humagne Rouge

Johanniter

Marsanne Blanche / Ermitage

Merlot

Müller-Thurgau / Riesling-Silvaner

Muscat / Muskateller

Petite Arvine

Pinot Blanc / Weissburgunder

Pinot Gris / Malvoisie / Grauburgunder

Pinot Noir / Blauburgunder

Räuschling

Regent

Sauvignon Blanc

Savagnin Blanc

Solaris

Sylvaner / Rhin

Syrah

Viognier

Add variety manually: ___________________

Add variety manually: ___________________

Add variety manually: ___________________

Add variety manually: ___________________

Add variety manually: ___________________

Add variety manually: ___________________

Add variety manually: ___________________

Add variety manually: ___________________

Add variety manually: ___________________

Add variety manually: ___________________

Add variety manually: ___________________

Add variety manually: ___________________

Add variety manually: ___________________

Add variety manually: ___________________

Add variety manually: ___________________

C3*. Please indicate the cultivated area per variety in **ares** below.

[Selected variety]: ________

[Selected variety]: ________

[Selected variety]: ________

**Section 2: Planting and management decisions**

D1. Please tick the box if the statement applies to you.

I am aware of fungus-resistant grapes

I have evaluated the overall potential (e.g. production, marketing, etc.) of cultivating fungus-resistant grapes on my farm

D2. [If above is ticked]

- The potential of red fungus-resistant varieties is [0-5]

- The potential of white fungus-resistant varieties is [0-5]

I have tried growing fungus-resistant grapes on parts of my land

I grow fungus-resistant grapevine varieties I will do that in the future too

I used to grow fungus-resistant grapes but stopped doing so.

D3. [If above is ticked]

- Why did you stop growing fungus-resistant varieties?

D4*. How much do you think fungus-resistant grapes reduce fungicide applications **on average** in Switzerland?

No fungicide applications are needed anymore (100%)

By 75-99%

By 50-74%

By 25-49%

By 1-24%

They do not reduce fungicide applications (0%)

I do not know

D5*. In **10 years**, what proportion of your grapevines will be replanted (in percentage)?

D6*. How much of your land will you devote to fungus-resistant varieties in **10 years**?

Less than today

About the same as today

More than today

I do not know

D7*. What proportion of your vineyard do you believe will be devoted to **fungus-resistant varieties** in **10 years** time?

- The most likely proportion of land devoted will be: ____________%
- The smallest possible proportion of land devoted will be: ____________%
- The largest possible proportion of land devoted will be: ___________%

D8*. Which factors do you consider to have the biggest negative impact on your grapevine **yield** (quality and quantity)?

Hail damage

Fungal infections

Insect infestations or pest infestations

Droughts

Weeds

Frost

Other: ___________

D9*. How do you control for **weeds** in your vineyard?

Mechanical weeding

Mulching

Herbicides

Other: _____________

D10*. How do you control for **insect infestations** in your vineyard?

Confusion techniques (e.g. Pheromones)

Promotion of beneficial insects (e.g. predatory mites, beetles)

Preventive measures (e.g. Field hygiene, irrigation, plant nutrition)

Decision support tools (e.g. Early warning systems, prognosis systems, damage threshold systems)

Insecticides

Mechanical control (e.g. Nets, traps)

Non-chemical plant protection products (e.g. Pyrethrin, spinosad, kaolin, oils, acids, etc.)

Other: __________

D11*. How do you control for **fungal infections** in your vineyard?

Remove infected material from the vineyard

Decision support tools (e.g. Early warning systems, prognosis systems, damage threshold systems)

Canopy management (e.g. thinning of clusters, air flow control, leaf removal)

Use of microorganisms (e.g. Bacillus subtilis, B. pumilus, Trichoderma spp., Fusarium spp)

Control of fertilizer

Use of inorganic material (e.g. Potassium bicarbonate, Ulmasud, Myco-Sin and Myco-San)

Fungicides

Other: _________

D12*. Below are **10 groups** of fungicides used in grapevine growing in Switzerland.

*Hint: Please indicate whether you use any of the products from the 10 groups. If you do not use any of them, please select the option "I do not use any of the fungicides shown above".*

Airone / Biorga Contra Kupfer Duo

Biorga Contra Kupfer / Capito Cupro / CUPROFIX 35 / Cupromaag 35 / Gesal Tomaten-Pilzschutz Vitigran / Oxykupfer 35 / Vitigran 35

Bordeaubrühe WG - Bouillie bordelaise WG / Bordeaux S / Bouillie bordelaise RSR / Kupfer-Bordo LG (Bouillie bordelaise LG) / Super bouillie Macclesfield 80

Bouillie bordelaise

Fluidosoufre

Gesal Kupfer-Pilzschutz

Kumulus WG / Thiovit Jet

LBG-42FFm

Netzschwefel Stulln

Oxychlorure de cuivre

I do not use any of the fungicides shown above

D13. Please indicate how many different products you use in the shown group(s):

Airone / Biorga Contra Kupfer Duo: _________

Biorga Contra Kupfer / Capito Cupro / CUPROFIX 35 / Cupromaag 35 / Gesal Tomaten-Pilzschutz Vitigran / Oxykupfer 35 / Vitigran 35: ________

Bordeaubrühe WG - Bouillie bordelaise WG / Bordeaux S / Bouillie bordelaise RSR / Kupfer-Bordo LG / Bouillie bordelaise LG / Super bouillie Macclesfield 80: ___________

Kumulus WG / Thiovit Jet : __________

E1. [control]

E2. [General information] Based on a scientific risk assessment for plant protection products, which considers the **environmental side effects** (i.e. persistence in soil / biomass as well as toxicity to non-target organisms) but also the product formulation and application rates, **22 fungicides** (5% of all fungicides authorized in Swiss viticulture) are considered to pose a **high risk to the environment**.

E3. [Personal information] Based on a scientific risk assessment for plant protection products, which considers the **environmental side effects** (i.e. persistence in soil / biomass as well as toxicity to non-target organisms) but also the product formulation and application rates, **22 fungicides** (5% of all fungicides authorized in Swiss viticulture) are considered to pose a **high risk to the environment**.

Based on the your previous answers, you use **[NUMBER_OF_PRODUCTS]** of these fungicides.

**Section 3. Information about the farm manager**

F1. What is your gender?

Female

Male

F2*. In which year were you born? _________

F3*. Do you have formal training in agriculture/viticulture from any of the following?

EFZ (e.g. Farmer, Winzer, Weintechnologe)

Agricultural management school/ "Meister" degree

Higher technical school (HF)

University of applied science

University or ETH

Fachbewilligungskurs Pflanzenschutz

Further education in plant protection

Other: ________

F4. Do you have a successor to your farm?

Yes

Rather yes

Rather no

No

No, there is no farm succession in the next 15 years

No, the farm will be discontinued

F5*. Do you agree with the following statements?

|  | Strongly  disagree | Disagree | Neither  agree nor  disagree | Agree | Strongly  agree |
| --- | --- | --- | --- | --- | --- |
| My main expertise is in grapevine production |  |  |  |  |  |
| My main expertise is in vinification |  |  |  |  |  |
| My main expertise is in marketing |  |  |  |  |  |

F6. What task/tasks do you complete at the vineyard?

Field work

Plant protection

Office work (e.g. sales, book keeping, etc.)

Planting decisions

Investment decisions

Vinification

Other: _________

F7. Which percentage of your earning are from farming?

0%

1-25%

26-50%

51-75%

76-100%

F8. How much is viticulture contributing to your farming income (in percent)?

0%

1-25%

26-50%

51-75%

76-100%

F9. Please rank these goals regarding their importance for your decisions on the farm (7 = highest importance, 6, 5, 4, 3, 2, 1 = lowest importance).

| Your options | Your ranking |
| --- | --- |
| High income  High yields  High grape quality for wine-making  Balancing work and leisure time  Social acknowledgement  Customer satisfaction  Environmental stewardship |  |

F10*. By the time you are **[CURRENT_AGE_+_10] years** old ...

... you will not work in viticulture anymore

... you will grow grapevines organically

... new technologies will allow reduced fungicide use (e.g. yeasts)

... weather events will increase fungi pressure in Swiss agriculture

... copper will be a banned substance in Swiss viticulture

... fungicide resistance will be a large issue

None of the above

F11*. By the time you turn **[CURRENT_AGE_+_10] years** old, how much of your land will you devote to **traditional vitis vinifera** varieties?

Less than today

The same as today

More than today

I do not know

F12*. By the time you are **[CURRENT_AGE_+_10] years** old, what share of your vineyard do you believe will be devoted to **traditional/non-resistant grapevine** varieties?

- I will most likely have: _______%
- I will not have less than: ________%
- I will not have more than: _________%

**Section 4: Information about the farm**

G1. How are you marketing your grapevines?

As grapes

As wine

G2. [As grapes] How are you marketing grapes (in percent)?

Sales to winemakers: _________%

Sales to cooperatives: _________%

Sales to commerce: _________%

G3. [As wine] How are you marketing your wines (in percent)?

Direct marketing: ________

Sales to commerce: _______

Sales to major distributors: ________

Sales to gastronomy: ________

G4*. Do you sell your wine under any of the following **labels** or **terms**?

Organic

Demeter

Vinatura

Natural

Delinat

PIWI

IP Suisse

None

Other: _______

G5*. Do you produce your wine under AOC/DOC?

Yes

No

G6. [Yes] Please let us know which varieties you market under AOC/DOC?

[VARIETY]:  Yes,  No

**Section 4: Information about the farm**

H1*. To link your answers with weather data from your region, please fill in your post code below: ______

H2. What is the focus of your farm? You can only choose one focus of production.

Viticulture

Orcharding and viticulture

Stone fruit production (cherries/plums/apricots))

Pome production (apples/pears)

Pome and stone fruit production

Mixed farm; viticulture and livestock farming

Other

H3*. According to which production from do you manage your farm?

ÖLN/VITISWISS

Organic

Integrated production (IP-Suisse)

Bio-dynamic

Other

H4. How many units of standard manpower does your farm have? _______

H5. What share of your farmland are you leasing?

0-25%

26-50%

51-75%

76-100%

H6. Which of the following strategies are your employing?

Agriculture-related diversification on farm (e.g. agrotourism)

Working off-farm (e.g. consulting, tourism)

Creation of financial reserves (saving for bad times)

Off-farm investments (e.g. other companies, real estate)

Processing and direct marketing

Forestry work

Other

H7. How important is (farm) biodiversity to you?

Not at all important

Not important

Neutral

Important

Very important

H8. Do you consider biodiversity decline on your farm an issue for your production?

Yes

No

H9. Do you use greening of inter-row space at your farm?

Yes

No

H10. [Yes] Do you consider or use (flowering) species-rich seed mixtures for greening inter-row space?

Yes

No

H11. What direct payment programmes does the farm participate in for vines?

M1: Partial abandonment of herbicides

M2: Complete abandonment of herbicides

M3: Abandonment of fungicides with particular risk potential, reduction of copper

M4: Abandonment of fungicides with particular risk potential, no use of copper

None

Other

H12. How are plant protection products applied on vines? Please tick all methods used on the vineyard.

Hand sprayer

Tractor

Closed-cabin tractor

Low drift nozzle

Spraying equipment with horizontal air assistance

Gun

Tunnel recycling sprayer

Helicopter

Drone

Other

H13. Have you received direct payments for the following purchases/investment of application methods?

25% acquisition cost for spray blower with horizontal air flow control

25% acquisition cost for spray blower with horizontal air flow control and vegetation dector

None of the above

H14. Where do you search for your plant protection information?

Via internet

Cantonal information services (e.g. Kantonale fachstellen, Kompetenzzentren)

Social media (e.g. Facebook, Twitter…)

Agroscope

Fibl

Other farmers/colleagues

I do not search for new information

Other

**Section 5: Fungus-resistant grapes**

I1*. How strongly do you agree or disagree with the following statements about fungus-resistant grapes?

|  | Strongly  disagree | Disagree | Neither agree or  disagree | Agree | Strongly  Agree |
| --- | --- | --- | --- | --- | --- |
| Wine from fungus-resistant varieties is of lower quality than  traditional varieties |  |  |  |  |  |
| Wine from fungus-resistant varieties is difficult to market |  |  |  |  |  |
| Consumers are willing to pay less for wine from fungus-resistant  varieties |  |  |  |  |  |
| Fungus-resistant wine use will increase in the future |  |  |  |  |  |
| Compared to traditional varieties fungus-resistant varieties are  better for the environment |  |  |  |  |  |
| Compared to traditional varieties fungus-resistant varieties have a  positive impact on the human health of farmers and communities surrounding farms |  |  |  |  |  |

I2*. How much do you know about fungus-resistant varieties?

| 0 (Nothing) | 1 | 2 | 3 | 4 | 5 (very knowledgeable) |
| --- | --- | --- | --- | --- | --- |
|  |  |  |  |  |  |

I3*. Have you looked for information about the cultivation of fungus-resistant varieties?

Yes

No

I4. [Yes] Where have you searched for this information?

Via internet (google, etc.)

Canton information services (e.g. Kantonale Fachstellen, Kompetenzzentren, etc.)

Social media (e.g. Facebook, Twitter, etc.)

Agroscope

Fibl

Farmers who grow fungus-resistant varieties

Organic grapevine producers

Communication with Valentin Blattner

PIWI association

Vine nursery

Other: ___________

I5. [No] Why not?

I do not know what fungus-resistant varieties are

I will never grow fungus-resistant varieties

I don’t trust the resistance of fungus-resistant varieties

I know already the advantages/disadvantages of fungus-resistant varieties

Other

I6. What are your experiences / opinions with growing fungus-resistant grape varieties? ____________

**Section 6: Perceptions and preferences**

J1*. How willing are you to give up income that is beneficial for you/the farm today in order to benefit more from that in the future?

| 0  Not willing | 1 | 2 | 3 | 4 | 5 | 6 | 7 | 8 | 9 | 10  Very willing |
| --- | --- | --- | --- | --- | --- | --- | --- | --- | --- | --- |
|  |  |  |  |  |  |  |  |  |  |  |

J2*. Are you willing to take risks or do you try to mitigate risks in the areas mentioned below?

Please indicate on the scale below. The value 0 stands for "not willing to take a risk at all" and the value 10 stands for "very willing to take a risk". With the values in between you can grade your assessment.

|  | 0  Not willing | 1 | 2 | 3 | 4 | 5 | 6 | 7 | 8 | 9 | 10  Very willing |
| --- | --- | --- | --- | --- | --- | --- | --- | --- | --- | --- | --- |
| Production |  |  |  |  |  |  |  |  |  |  |  |
| Market and prices |  |  |  |  |  |  |  |  |  |  |  |
| Plant protection |  |  |  |  |  |  |  |  |  |  |  |
| Agriculture in general |  |  |  |  |  |  |  |  |  |  |  |

**Section 6: Perceptions and preferences**

K1. How strongly do you agree or disagree with the following statements about plant protection products?

|  | Strongly  disagree | Disagree | Neither  agree or  disagree | Agree | Strongly  agree |
| --- | --- | --- | --- | --- | --- |
| Plant protection products have a positive effect on wine quality |  |  |  |  |  |
| Plant protection products have a negative effect on soil |  |  |  |  |  |
| Plant protection products have a positive effect on wine quantity |  |  |  |  |  |
| Plant protection products have a negative effect on the environment |  |  |  |  |  |
| Plant protection products have a negative effect on the farmers' health |  |  |  |  |  |

K2. What influence does ...

|  | Strongly  disagree | Disagree | Neither  agree or  disagree | Agree | Strongly  agree |
| --- | --- | --- | --- | --- | --- |
| ... my grapevine production have on the environment? |  |  |  |  |  |
| ... my grapevine production have on the human health of communities surrounding farms? |  |  |  |  |  |
| ... my grapevine production have on my own health? |  |  |  |  |  |
| ... my grapevine production have on maintaining cultural |  |  |  |  |  |

K3. Do you agree or disagree with the following statements about your attitudes towards grape production?

|  | Strongly  disagree | Disagree | Neither  agree or  disagree | Agree | Strongly  agree |
| --- | --- | --- | --- | --- | --- |
| When I encounter difficulties in grape/wine production, I can usually think of a solution |  |  |  |  |  |
| I am confident that I can accomplish my production goals at the end of the harvest |  |  |  |  |  |
| I can solve production issues if I invest the necessary effort |  |  |  |  |  |
| How successful my grape/wine production is depends mostly on my skills as a farmer |  |  |  |  |  |
| Grapevine growing is more dependent on the weather than on what I do |  |  |  |  |  |
| Success in grapevine production can only be slightly influenced by farmers |  |  |  |  |  |
| I usually set myself quite ambitious production goals |  |  |  |  |  |

**Section L: Concluding questions**

L1. For your farm, was the last growing year different compared to the last 5 growing years? If yes, how? _______________

L2. Do you have any comments or feedback in general? ________________

L3. You said yes to receiving the results of the survey and/or entering the draw for the **50 CHF Landi vouchers**, please let us know your email address so we can send you these results: _______________

**Thank you very much for your participation**.

We will treat your personal data with strictly confidential. If you have any questions or comments please contact:

XXXXX
XXXXX
XXXXX

If you left us your e-mail address and asked for individual feedback, we will send you feedback. If you are the lucky winner of the draw, we will notify you of course.

**References**

[1] L. Zachmann, C. McCallum, R. Finger, Nudging farmers towards low‐pesticide practices: Evidence from a randomized experiment in viticulture, J of Agr & App Econ Assoc. 2 (2023) 497–514. https://doi.org/10.1002/jaa2.76.

[2] O. Viret, J.-L. Spring, V. Zufferey, K. Gindro, C. Linder, A. Gaume, F. Murisier, Past and future of sustainable viticulture in Switzerland, BIO Web Conf. 15 (2019) 01013. https://doi.org/10.1051/bioconf/20191501013.

[3] T. Dohmen, A. Falk, D. Huffman, U. Sunde, J. Schupp, G.G. Wagner, Individual Risk Attitudes: Measurement, Determinants, and Behavioral Consequences, Journal of the European Economic Association. 9 (2011) 522–550. https://doi.org/10.1111/j.1542-4774.2011.01015.x.

[4] A. Falk, A. Becker, T. Dohmen, B. Enke, D. Huffman, U. Sunde, Global Evidence on Economic Preferences, The Quarterly Journal of Economics. 133 (2018) 1645–1692. https://doi.org/10.1093/qje/qjy013.

[5] L. Knapp, E. Bravin, R. Finger, Data on Swiss fruit and wine growers’ management strategies against D. suzukii, risk preference and perception, Data in Brief. 24 (2019) 103920. https://doi.org/10.1016/j.dib.2019.103920.

[6] Bundesamt für Statistik (BFS), Soziale Aspekte, (2022). https://www.bfs.admin.ch/bfs/de/home/statistiken/land-forstwirtschaft/landwirtschaft/soziale-aspekte.html (accessed August 22, 2022).

[7] C. Widmer, Agrarbericht 2021 - Betriebe, (2021). https://www.agrarbericht.ch/de/betrieb/strukturen/betriebe (accessed August 22, 2022).

[8] Bundesamt für Landwirtschaft (BLW), Das Weinjahr 2021, 2022. https://www.blw.admin.ch/dam/blw/de/dokumente/Nachhaltige%20Produktion/Pflanzliche%20Produktion/Weine%20und%20Spirituosen/Weinwirtschaftliche%20Statistik/weinjahr_2021.pdf.download.pdf/d_weinjahr_2021.pdf (accessed August 5, 2022).

[9] P.-H. Dubuis, B. Bloesch, A.-L. Fabre, O. Viret, Lutte contre l’oïdium à l’aide du modèle VitiMeteo-Oidium, Revue Suisse Viticulture, Arboriculture, Horticulture. 46 (2014) 368–375.

[10] P.H. Dubuis, G. Bleyer, R. Krause, O. Viret, A.-L. Fabre, M. Werder, A. Naef, M. Breuer, K. Gindro, VitiMeteo and Agrometeo: Two platforms for plant protection management based on an international collaboration, BIO Web Conf. 15 (2019) 01036. https://doi.org/10.1051/bioconf/20191501036.

[11] K. Schroeer, S. Trefalt, C. Schwierz, A. Hering, U. Germann, CHHC: Swiss Hail Climatology, (2021). https://doi.org/10.18751/CLIMATE/GRIDDATA/CHHC/1.0.

[12] MeteoSchweiz, Räumliche Analysen der langjährigen Monats- und Jahresmittel der relativen Sonnenscheindauer in der Normperiode 1961-1990, 2020. https://www.geocat.ch/geonetwork/srv/ger/catalog.search#/metadata/e7873de9-c1dc-4ded-a417-2c4dc2d839fc (accessed August 27, 2021).

[13] P. Kudsk, L.N. Jørgensen, J.E. Ørum, Pesticide Load—A new Danish pesticide risk indicator with multiple applications, Land Use Policy. 70 (2018) 384–393. https://doi.org/10.1016/j.landusepol.2017.11.010.

[14] A. Weersink, M. Fulton, Limits to Profit Maximization as a Guide to Behavior Change, Applied Economic Perspectives and Policy. 42 (2020) 67–79. https://doi.org/10.1002/aepp.13004.

[15] A. Carbone, L. Quici, G. Pica, The age dynamics of vineyards: Past trends affecting the future, Wine Economics and Policy. 8 (2019) 38–48. https://doi.org/10.1016/j.wep.2019.02.004.

[16] H.A. Simon, Bounded Rationality, in: J. Eatwell, M. Milgate, P. Newman (Eds.), Utility and Probability, Palgrave Macmillan UK, London, 1990: pp. 15–18. https://doi.org/10.1007/978-1-349-20568-4_5.

[17] J.B. Rotter, Generalized expectancies for internal versus external control of reinforcement, Psychological Monographs: General and Applied. 80 (1966) 1–28. https://doi.org/10.1037/h0092976.

[18] A. Bandura, Guide for constructing self-efficacy scales, in: F. Pajares, T.C. Urdan (Eds.), Self-Efficacy Beliefs of Adolescents, IAP - Information Age Pub., Inc, Greenwich, Conn, 2006.

[19] L. Knapp, D. Wuepper, R. Finger, Preferences, personality, aspirations, and farmer behavior, Agricultural Economics. (2021) agec.12669. https://doi.org/10.1111/agec.12669.

[20] K.A. Abay, G. Blalock, G. Berhane, Locus of control and technology adoption in developing country agriculture: Evidence from Ethiopia, Journal of Economic Behavior & Organization. 143 (2017) 98–115. https://doi.org/10.1016/j.jebo.2017.09.012.

[21] T. Tanaka, C.F. Camerer, Q. Nguyen, Risk and Time Preferences: Linking Experimental and Household Survey Data from Vietnam, American Economic Review. 100 (2010) 557–571. https://doi.org/10.1257/aer.100.1.557.

[22] J.B. Hardaker, G. Lien, J.R. Anderson, R.B.M. Huirne, Coping with risk in agriculture: applied decision analysis, Third edition, CABI, Boston, MA, 2015.

[23] N. Möhring, R. Finger, P. Kudsk, L. Vidensky, L. Nistrup Jørgensen, J.E. Ørum, PesticideLoadIndicator (R-Package), (2021). https://doi.org/10.3929/ETHZ-B-000488621.

[24] A.J. Berinsky, G.A. Huber, G.S. Lenz, Evaluating Online Labor Markets for Experimental Research: Amazon.com’s Mechanical Turk, Polit. Anal. 20 (2012) 351–368. https://doi.org/10.1093/pan/mpr057.
